# Graph-based process models as basis for efficient data-driven surrogates – expediting the material development process

**DOI:** 10.1016/j.csbj.2025.04.018

**Published:** 2025-04-24

**Authors:** Johannes Gerritzen, Andreas Hornig, Maik Gude

**Affiliations:** a Institute of Lightweight Engineering and Polymer Technology (ILK), TUD Dresden University of Technology, Holbeinstr. 3, Dresden, 01307, Germany; b Center for Scalable Data Analytics and Artificial Intelligence Dresden/Leipzig (ScaDS.AI), TUD Dresden University of Technology, Chemnitzer Str. 46b, Dresden, 01187, Germany; c Department of Engineering Science, University of Oxford, Parks Road, Oxford, OX1 3PJ, United Kingdom

**Keywords:** Process development, Surrogate modeling, Data-driven decision making

## Abstract

Shorter development cycles, increasing complexity and cost pressure are driving the need for more efficient development processes. Especially in the field of material development, the long and costly experiments are a major bottleneck. To address this bottleneck, data-driven models supporting the decision making process have recently gained popularity. However, such models require a structured representation of the development process to allow an efficient training. In this work, a formalism for deriving an efficient representation of material development processes (MDPs) is proposed, and demonstrated on the development of a high modulus steel (HMS). The formalism is based on the combination of graph-based process models and the recently proposed concept of “flowthings” [1]. This allows to efficiently derive a directed acyclic graph (DAG) representation of the MDP with the acquired data. From this, a database for subsequent training of surrogate models is derived, on which several black box models for the MDP are trained. Best-in-class models are chosen based on the root mean squared error (RMSE) on the test set and subsequently used for the inverse optimization of the MDP to maximize the specific modulus while meeting additional design constraints. This showcases the potential of the proposed formalism to accelerate the MDP through data-driven modeling.

## Graphical abstract

**Figure:**
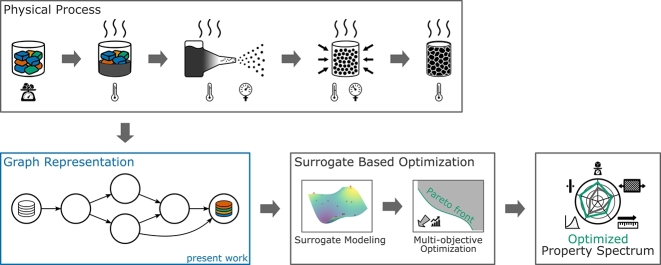

## 1 Introduction

Increasing environmental and economical requirements accelerate the development of novel technologies in the aviation industry [2]. This led to a significant growth in aircraft turbines overall but especially their bypass ratios [3]. The resulting loads, which the turbine has to withstand, are so high that conventional materials cannot be used anymore.

Hence, novel steel alloys with significantly increased specific Young's modulus, so called HMS, are being developed for the application in high bypass ratio turbines. One approach that is being thoroughly investigated since 25 years is the formation of microscopic ceramic particles that are embedded in the metallic matrix [4], [5], [6], [7]. Especially the in-situ formation of the reinforcement particles is highly desirable, since it allows the reduction of the number of process steps required up to the final product. This, however, introduces additional complexity, which, despite the substantial research effort, is still the focus of current research [8], [9].

The final material properties depend on the entire upstream process chain. Hence, achieving a profound understanding necessitates the consideration of the entirety of involved process steps. The consistent monitoring and modeling of processes has been a promising approach to capture influence and inherent interdependencies of the plethora of governing parameters. Based on the structured foundations of product development processes, i.e. summarized in [10], and the principle of an interactive design process [11], process models have been proposed by many authors.

Bowers et al. developed a model to establish data provenance which leverages queries over logs to obtain the entire history of a data item post hoc [12]. From this, they derive dependency graphs for the specific process leading to the data item at hand. Khodabandelou et al. point out the importance of taking the intention behind the modeling effort into account from the beginning to significantly enhance the efficiency of the modeling process [13]. They leverage the intent for automated process mining from logged information, leading to a fine-grained representation of the observed processes which is coarse-grained in a second step, introducing a higher level of abstraction. Extending classical methodologies, Zhang et al. proposed the “innovative design model” as a system engineering base that combines static requirement analyses with dynamic response capabilities [14]. Dolean and Petrusel highlight a shortcoming of many works that focus purely on control structures and the physical process itself, neglecting the data flow [15]. They showcase that capturing the flow of data throughout the process is crucial for a comprehensive understanding and the model's applicability.

Schabacker et al. streamlined the formalism of business process model and notation, a design structure matrix and a container model into one coherent solution supporting the optimization of modeled processes [16]. Bruno built on this by extending the formalism to a dataflow-oriented modeling approach [17]. Jin and Liu proposed an extension of process monitoring, and thus the basis of its modeling, by introducing control charts for multistep processes with inherent parallelisms [18]. Al-Fedaghi proposed an alternative to the business process model and notation formalism by introducing so-called “flowthings”, general objects that are passed from one process step to the next [1]. In each step, the flowthing is processed and its state is updated based on the actions taken on it. This enables an efficient transfer of data along the process chain. To modularize process representation, Haider et al. subdivided process steps into model, method and data, thus defining a clear interface between individual process steps [19]. Stanković et al. proposed the representation of process chains by directed multigraphs enabling the integration of expert knowledge through custom graph grammar rules [20]. Van Hee et al. elucidate how dependencies in processes can be modeled using Petri Nets [21]. This was later extended by Medina-Garcia et al. by introducing the concept of so called basic structures into Petri Nets, facilitating their understanding and application [22].

To further deepen the understanding of processes and allow for efficient evaluations of parameter adaptations, linked simulations have become an invaluable tool. Fürstenau et al. used smooth particle hydrodynamics simulations to obtain a virtual process map of a selective laser melting process [23]. This was necessary due to the high dimensionality of the parameter space the selective laser melting process spans. Similarly, Vohra et al. used a combination of simulation data and dimensionality reduction techniques as the basis for surrogate model training for best performance and sensitivity analyses [24]. Flowsheet simulations offer a powerful tool for physically based modeling of complex, interacting processes. However, they are often limited to modeling processes and typically exclude key properties of the final product [25]. Skorych et al. showcased how a data-driven model can be integrated into flowsheet simulations to allow for an efficient analysis of varying process parameters [26]. Zitzewitz and Fieg demonstrated how a reliable process model can enable the optimization of the underlying process and thus deepen the understanding thereof [27]. Hürkamp et al. ran detailed process simulations of thermoforming followed by overmolding processes to gain detailed insights and generate a reliable data foundation. The results were trained into surrogate models, which were in turn used to transfer the gained knowledge to the operational phase [28]. Pfrommer et al. optimized processes using artificial neural networks (ANNs) as surrogate models [29] via iterative training on simulation data.

In recent years, the use of data-driven models to tackle complex tasks and to save on expensive experiments or simulations has become increasingly popular in the fields of engineering and materials science. Tao et al. used Gaussian processes (GPs) to optimize vehicle suspension for dynamic stability [30]. Sun et al. compiled a review on the application of ANNs as surrogate model in the design of aerodynamic parts [31]. Hadidi et al. used a response surface model for reliability analysis in high dimensional spaces [32]. Teng et al. approached a similar challenge by training a model capable of assessing a system's reliability by applying generative adversarial theory to surrogate modeling [33]. Yan et al. employed a combination of GPs and physically based methods to predict the mechanical properties of additively manufactured alloys based on their composition and process parameters [34]. Leveraging accurate forward models, significant research effort aims to invert this approach and utilize the inherent knowledge in order to adapt the process to achieve desirable results. Jiang et al. used surrogate models for the optimization of designs [35]. Gerritzen et al. proposed a constitutive model for the out of plane shear behavior of fiber reinforced polymers derived from pure data analysis [36]. They further facilitated its usage through an ANN based method for direct parameter identification, capable of directly extracting material parameters from experimental stress-strain curves [37].

**Fig. 1:**
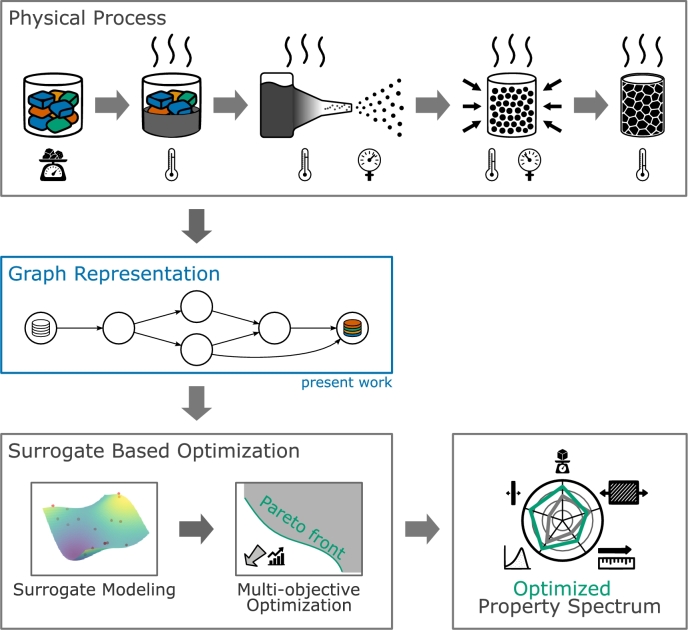
Domain knowledge enhanced representation of physical process as DAG as foundation for surrogate based optimization of product properties.

So far, many approaches to model interdependencies in processes have been proposed in the literature. However, the majority of these approaches focuses exclusively on either the physical process or the data flow, but rarely both. In this work, we propose a graph-based framework enhanced by domain knowledge that allows for a unified representation of both the physical process and the data flow, laying the groundwork for efficient model integration. This is achieved by incorporating domain knowledge to establish a formalism representing the process chain as a DAG, where nodes represent process steps and edges represent their connection and flow of material, data and information between them. The proposed framework thus allows for a more streamlined representation of the process, enabling data-based optimization and decision-making. Its fundamental role as connecting link between physical process and data-based optimization is illustrated in Fig. 1.

The framework's capabilities are demonstrated by modeling a real world material manufacturing process for an HMS, which consists of multiple steps. The aim is to transfer influential parameters and a process map into a representation that can be used for subsequent training of a surrogate model. For this, several models are compared and the “best-in-class” version is chosen for final analyses. The chosen model is used for the efficient optimization of the overall process objective, i.e. maximizing specific stiffness while meeting other design allowables. This is carried out using the NSGA-II algorithm [38] to obtain a candidate set of process parameters expected to yield an excellently performing material that should be tested next.

## 2 Formal process model

**Fig. 2:**
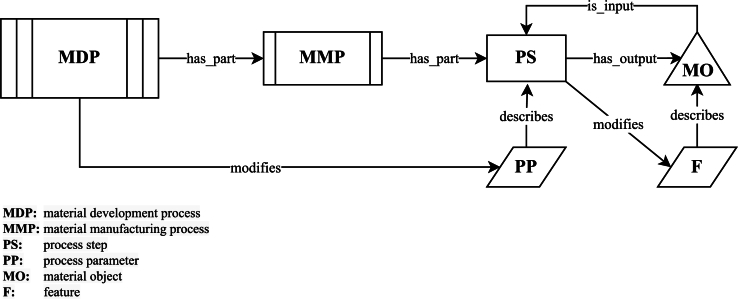
Relationships of material development process and its constituents.

To efficiently capture and link all relevant data with their respective meta-data along the development process, the “flowthing” approach from [1] is adapted to the MDP. Specifying the “flowthing” in this context, the final material as well as its precursors are collected under the term material object. Each material object is characterized by its current features and labeled with a unique identifier. In process steps, the material object's features are changed and augmented, based on the present process parameters, representing the material's evolution throughout the process. The succession of multiple process steps that lead to a finished material are represented by a DAG, with the process steps characterized by a concrete set of process parameters as its nodes and the material object being passed along its edges. The resulting heterogeneous graph constitutes one material manufacturing process (MMP). All MMPs that are carried out until design goal is met, combined with the reasoning behind respective changes of process parameters across different MMPs, comprise the MDP. The formalized relationships are illustrated in Fig. 2.

The formal relationships lay the foundation for a general database scheme capable of storing data from diverse processes and sources. To ensure a lean database, it is possible to reduce the tracked process parameters in this step based on expert knowledge on the process on hand.

From the proposed formalism, three approaches for establishing a surrogate model of the process of interest can be followed:

**Step-wise modeling:** A process-step-based approach in which property changes caused by each process step are modeled explicitly and are propagated to the next process step. This enables the targeted use of physically based models, where expert knowledge on underlying relationships exists, allowing for deep process insights and yielding validation possibilities along the process chain. Individual steps that cannot be captured by physics-based models can be substituted by data-driven models. However, this requires detailed information on the current state of features after each process step, imposing substantial experimental effort on the MDP.
**Monolithic modeling:** A fully data-driven solution that aggregates all process parameters along the edges of the graph and correlates them directly with the final material properties. This requires the least amount of tests per MMP, but significantly limits the possibility of including expert knowledge into the process model.
**Hybrid modeling:** A compound approach, aggregating multiple process steps into one surrogate where necessary while leveraging preexisting knowledge and physically based models, where they are available. This enables a use-case-specific trade-off between modeling fidelity and efficiency.

Having such a surrogate model allows to quickly estimate how changes to the process will influence the resulting product. This can be used for inline process monitoring to check if deviations from the intended process parameters will be detrimental to the final features. Additionally, such a model may be used for accelerating the MDP by leveraging surrogate based optimization techniques, see i.e. [39]. The model's sequential evaluation with process parameters obtained from established optimization frameworks allows to quickly converge to an arbitrary objective or assess the Pareto front when multiple objectives have to be considered.

## 3 Investigation of the applicability to a real world MDP of HMS

In this work, a manufacturing process for Fe-Ti-B-Cu HMS adapted from [40] is used as example. Instead of liquid metallurgy and casting, a powder metallurgical approach in combination with a hot isostatic pressing (HIP) process for the creation of ceramic  particles is followed, as suggested in [41]. The final material is obtained by HIP and subsequent heat treatment. For the HMS, physical properties Young's modulus *E* and density *ϱ*, as well as mechanical properties yield strength (YS), ultimate tensile strength (UTS) and total elongation (TE) are of interest.

### 3.1 Building up graph representation of the MMPs

**Fig. 3:**
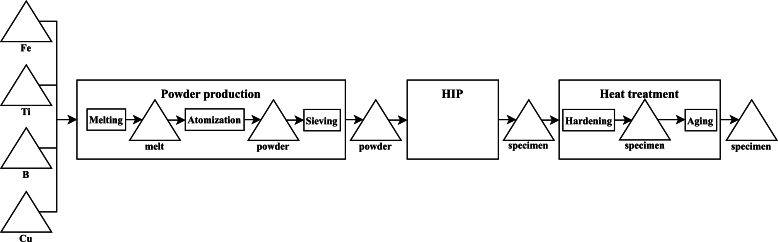
Graph representation of an MMP for HMS.

The powder production consists of the process steps melting, atomization and sieving. The HIP process is considered an atomic process step. The final product is obtained by heat treatment, consisting of hardening and aging. This leads to the graph representation shown in Fig. 3.

For the further analyses, powder production is not taken into account, since no data is available for the process parameters of the process step melting, atomization and sieving. Instead, the chemical composition of the powder is taken as adjustable input to the MMP.

### 3.2 Surrogate modeling of the MDP

Given the lack of data on changes in features throughout each MMP, a fully data-driven approach is chosen for the surrogate models. Hence, for each MMP all process parameters are accumulated along the edges of the DAG and jointly correlated with the corresponding features obtained from the final material object. For this, a total of 4 different chemical compositions were manufactured. These were subjected to different HIP and heat treatment conditions, leading to a total of 22 unique datapoints for the MDP, which will be used for training the surrogate models. However, the physical properties are expected to be purely dependent on the chemical composition. Hence, only 4 experimental datapoints exist for these features. To alleviate the lack of data for the physical properties, 25 datapoints for *E* obtained from simulation^2^ and for *ϱ* the 14 datapoints from literature given in Table A.1 are added.

**Fig. 4:**
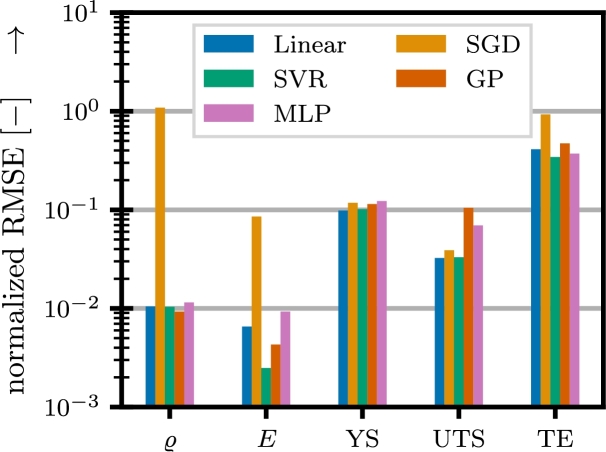
Comparison of the normalized RMSE obtained from investigated surrogate models evaluated on validation data.

Based on the available data, several surrogate models are investigated for predicting individual features from chemical composition and process parameters. Here, the following models from the open source Python library scikit-learn [42] are compared: 1. linear model, 2. stochastic gradient descent (SGD), 3. support vector regression (SVR), 4. GP and 5. multilayer perceptron (MLP). For each of them, except for the linear model, hyperparameter optimization is carried out using the hyperopt library [43]. To do this,  of the available data is withheld from training and used for validation. As an objective function for the hyperparameter optimization, the RMSE on the validation set is used. The resulting contamination of the validation data has to be accepted, since the available data is too sparse to allow for a separate test set. The RMSE normalized by the respective means of the features is shown in Fig. 4 for each model.

From this it becomes clear, that different models are better suited for different features. Especially for the physical properties, SGD performs significantly worse than the other models. For the mechanical properties, models show very similar performance. For the final analyses, the best-in-class model for each feature is chosen based on the lowest RMSE: 1. SVR for *E*, 2. GP for *ϱ*, 3. linear for YS, 4. linear for UTS and 5. SVR for TE. The corresponding hyperparameters are shown in Table 1 along with the achieved RMSE values.

**Table 1:** Hyperparameters and RMSE for the best-in-class surrogate models.

				Train		Test	
Property	Model	Hyperparameters		RMSE	*r*^2^	RMSE	*r*^2^
*E*	SVR	C	54.835	0.0198	0.95	0.0025	0.99
		epsilon	0.2974				
		degree	5				
		kernel	“RBF”				

*ϱ*	GP	kernel	“RationalQuadratic”	0.0109	0.70	0.0093	0.76
		alpha	1				
		length_scale	1				

YS	Linear	−		0.1300	0.59	0.0983	0.70

UTS	Linear	−		0.0472	0.83	0.0324	0.82

TE	SVR	C	12.241	0.2378	0.59	0.3436	0.40
		epsilon	0.1011				
		degree	4				
		kernel	“Linear”				

**Fig. 5:**
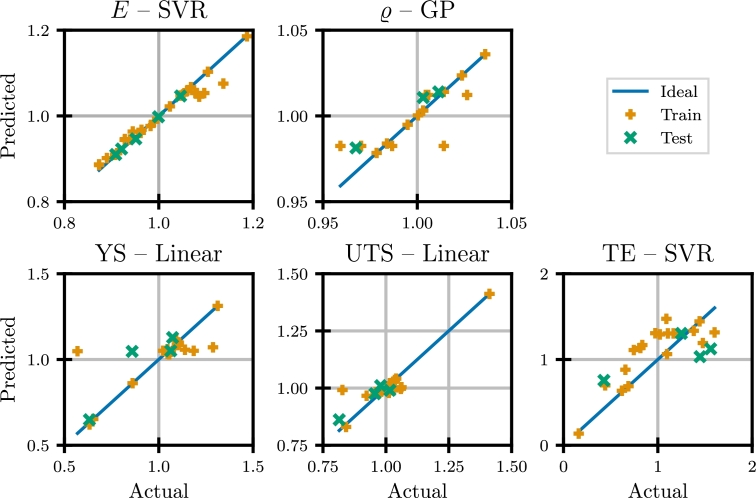
Comparison of actual and predicted values for the best-in-class HMS surrogate models on normalized values.

For these best-in-class models, the comparison of actual and predicted values is shown in Fig. 5 on normalized values. From this it becomes clear that the best-in-class models show overall very good performance. Especially for *E*, the SVR model excellently captures the behavior of the data, seamlessly integrating the simulation data. With *ϱ*, the GP model gives accurate predictions in spite of the literature data partly deviating from the simplifying assumption of a pure dependency on the chemical composition. The more complex strength based mechanical properties are also well captured by the models. For the YS, an $r^{2}$ score of 0.70 is achieved on the test set using the linear model. Similarly, the UTS is predicted with an $r^{2}$ score of 0.82. Solely for the TE, the SVR model shows a lower performance, with an $r^{2}$ score of 0.40. This can be attributed to the high variability of the data in combination with the comparatively low number of datapoints available for training.

### 3.3 Extraction optimal process parameters to maximize specific modulus of the HMS

To optimize the material's performance without additional experimental effort, the identified models are used. Based on them, the target of maximizing specific modulus, an objective is defined per O=Eϱ. In addition to that, several constraints from the design process have to be considered: for functionality, all mechanical properties have to exceed a certain threshold and to ensure the desired microstructure, the ratio of Ti and B has to be in a defined range.

**Fig. 6:**
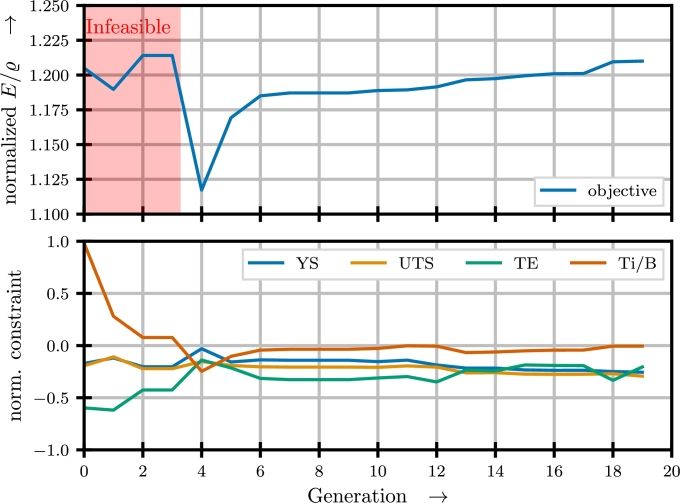
Results from optimizing chemical composition and process parameters for maximum specific modulus.

This problem statement is implemented as ElementwiseProblem using the open source Python library pymoo [44]. The actual optimization is carried out using the NSGA-II algorithm with a population size of 40 and 20 generations. The normalized results are shown in Fig. 6.

From this it becomes clear that initially, a very high specific modulus is predicted. However, this is achieved with a chemical composition leading to an undesirable microstructure. Throughout the optimization a viable candidate is quickly identified in generation 4, at a high cost of the objective. From that point forward, slight modifications to the candidates lead to a gradual improvement of the objective function whilst remaining at the verge of the feasible region of the chemical composition. Throughout the optimization, in each generation candidates not violating the constraints on mechanical properties are achieved. Based on this, a combination of chemical composition and process parameters is identified, that is predicted to have on par specific modulus with the best tested results, while meeting all design constraints.

## 4 Conclusion

To model MDPs and enhance them by data-driven guidance, two established concepts for general process modeling were combined: 1. graph based representation of processes and 2. attaching information to an object that is passed along the process. This gave rise to a formalism for representing MDPs, which allows to efficiently derive a DAG representation. The functionality has been shown exemplary on the MDP of an HMS. Based on the derived DAG and available features, a database for subsequent training of surrogate models was established. This database was extended using data from simulations and literature for the physical properties, since the assumption of them being only influenced by the chemical composition led to a severe reduction in available data.

Based on the database, a multitude of purely data-driven surrogate models for each feature were trained, each representing one entire MMP. To obtain the best possible model, during this stage a hyperparameter optimization was carried out for each investigated model class. From those the best-in-class model was chosen based on the RMSE on the test set for each feature respectively. The models showed good performance for all properties except the TE. Using the established models, a candidate of chemical composition and process parameters was identified that is predicted to lead to a significant improvement of the specific Young's modulus while meeting all design targets on the mechanical properties and ensures a favorable microstructure. This should be the next point in the sequential design.

## Acronyms

**ANN**: artificial neural network
**DAG**: directed acyclic graph
**GP**: Gaussian process
**HIP**: hot isostatic pressing
**HMS**: high modulus steel
**MDP**: material development process
**MLP**: multilayer perceptron
**MMP**: material manufacturing process
**RMSE**: root mean squared error
**SGD**: stochastic gradient descent
**SVR**: support vector regression
**TE**: total elongation
**UTS**: ultimate tensile strength
**YS**: yield strength

## Funding

The work presented in this paper was conducted within the framework of the MODUL research project (Federal Aviation Research Programme LuFo, German 10.13039/100021130Federal Ministry for Economic Affairs and Climate Action BMWK, Funding Reference: 20T1913A).

## Consent for publication

All authors have read the manuscript and agree with its publication.

## CRediT authorship contribution statement

**Johannes Gerritzen:** Writing – review & editing, Writing – original draft, Visualization, Validation, Software, Methodology, Investigation, Funding acquisition, Formal analysis, Data curation. **Andreas Hornig:** Writing – review & editing, Supervision, Resources, Project administration, Funding acquisition. **Maik Gude:** Writing – review & editing, Supervision, Project administration, Funding acquisition.

## Declaration of Competing Interest

The authors declare that they have no known competing financial interests or personal relationships that could have appeared to influence the work reported in this paper.

## Figures and Tables

**Table A.1:** Density values for HMS added from literature.

Alloy-system	density in	Reference
	7.32	[45]
	7.32	[45]
	7.3	[45]
	7.40	[8]
	7.59	[8]
	7.56	[46]
	7.08	[46]
()	7.2	[47]
()	7.0	[47]
()	7.4	[47]
	7.38	[48]
	7.4	[48]
	7.06	[40]
	7.26	[40]
	7.47	[40]
	7.54	[49]
	7.40	[49]
	7.43	[50]

## Footnotes

2 Simulations were carried out by QuesTek Innovations LLC.
